# Halftime rotational atherectomy: a unique concept for diffuse long severely calcified lesions

**DOI:** 10.1007/s12928-023-00968-1

**Published:** 2023-11-10

**Authors:** Kenichi Sakakura, Hiroyuki Jinnouchi, Yousuke Taniguchi, Takunori Tsukui, Yusuke Watanabe, Kei Yamamoto, Masaru Seguchi, Hideo Fujita

**Affiliations:** Division of Cardiovascular Medicine, Saitama Medical Center, Jichi Medical University, 1-847 Amanuma, Omiya, Saitama City, 330-8503 Japan

**Keywords:** Rotational atherectomy, Slow flow, Complications, Burr-to-artery ratio

## Abstract

Rotational atherectomy (RA) is technically more difficult in a diffuse calcified lesion than in a focal calcified lesion. We hypothesized that taking a halftime can be another option for RA to the diffuse calcified lesions. Halftime was defined as at least one long break during RA, in which an operator pulled out the Rotablator system from the guide catheter before crossing the lesion. This study aimed to compare the complications between RA with and without halftime. We included 177 diffuse long severely calcified lesions (lesion lengths ≥ 30 mm) that required RA, and divided those lesions into a halftime group (*n* = 29) and a no-halftime group (*n* = 148). The primary outcome was periprocedural myocardial infarction (MI). The reference diameter was smaller in the halftime group than in the no-halftime group [1.82 (1.70–2.06) mm versus 2.17 (1.89–2.59) mm, *p* = 0.002]. The total run time was longer in the halftime group than in the non-halftime group [133.0 (102.0–223.0) seconds versus 71.5 (42.0–108.0) seconds, *p* < 0.001]. Although creatinine kinase (CK) and CK–myocardial band (MB) was significantly higher in the halftime group than in the no-halftime group [CK: 156 (97–308) U/L versus 99 (59–216) U/L, *p* = 0.021; CK–MB: 15 (8–24) U/L versus 5 (3–15) U/L, *p* < 0.001], periprocedural MI was not observed in the halftime group. In conclusion, periprocedural MI was not observed in RA with halftime. This preliminary study suggests that halftime RA may be a safe option for diffuse severely calcified lesions.

**Graphical abstract:**

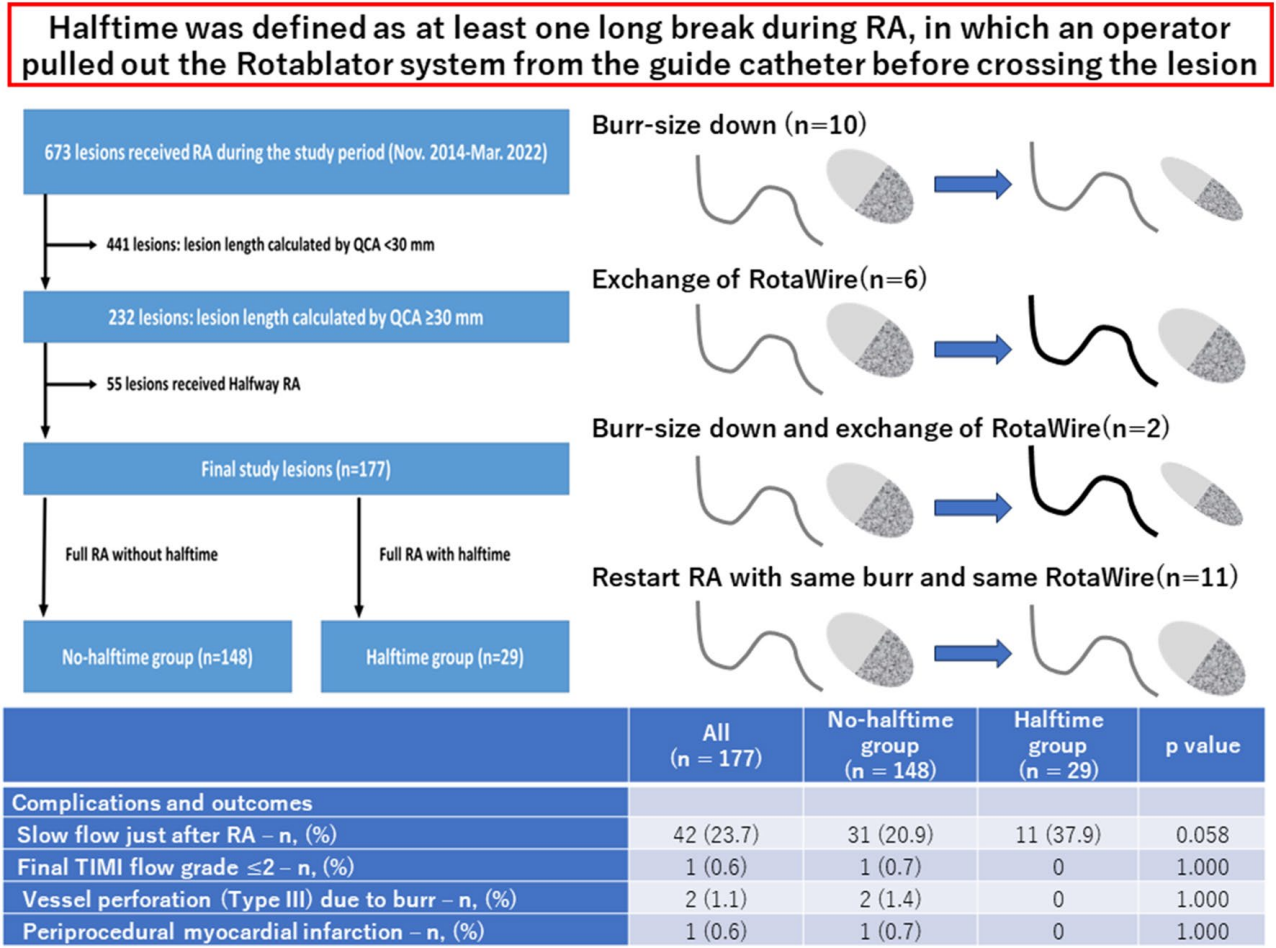

**Supplementary Information:**

The online version contains supplementary material available at 10.1007/s12928-023-00968-1.

## Introduction

Rotational atherectomy (RA) has been the cornerstone for the percutaneous coronary intervention (PCI) to severely calcified lesions [1–5]. Among severely calcified lesions, RA is technically more difficult in a diffuse long calcified lesion than in a focal short calcified lesion, because the lesion length is closely associated with the occurrence of complications during RA [6, 7]. The clinical expert consensus document on RA from the Japanese association of cardiovascular interventions and therapeutics (CVIT) proposes an algorithm for the diffuse long calcified lesions [8, 9]. In the algorithm, halfway RA is suggested if there is a steep angle within the lesion [10, 11]. However, when halfway RA is not applicable for the lesion, the options to ablate the whole diffuse long lesion are limited. The burr-size down or the exchange of RotaWire (Marlborough, MA, USA) is described in the algorithm [8, 9]. Moreover, the combination of the burr-size down and the exchange of RotaWire may be useful [12].

ST-segment elevation is common in RA to a diffuse long calcified lesion, and such ST-segment elevation reflects coronary slow flow, which varies from mild slow flow [thrombolysis in myocardial infarction (TIMI) flow grade 2] to severe slow flow (TIMI flow grade ≤ 1) [13, 14]. It may be important to take a sufficient break than to keep ablating the lesions without a break, when sustained ST-segment elevation is observed. Because the diameter of the drive shaft sheath of Rotablator (Boston Scientific, Marlborough, MA, USA) is 4.3 Fr (1.43 mm) [8, 9], it may be necessary to pull out the Rotablator system from the guide catheter to restore the sufficient coronary flow, which requires a long break like halftime rather than a short break between each session. We hypothesized that taking a long break (halftime) irrespective of changes of burr-size or RotaWire can be another option for RA to the diffuse long calcified lesions. The purpose of this study was to compare the clinical outcomes in RA to the diffuse severely calcified lesions between RA with and without halftime.

## Methods

This was a retrospective, single-center study. We reviewed 673 consecutive coronary lesions that were treated with RA in our institution during the period from November 2014 to March 2022. Indications for RA in our institution are the following: 1) angiographically moderately or severely calcified lesions, 2) diffuse lesions expected to be difficult to stent, and 3) ostial lesions [7, 15]. The primary outcome was periprocedural myocardial infarction (MI), which was defined as either an increase in creatine kinase–myocardial band (CK–MB) above 10 × upper limit normal (ULN) or CK–MB above 5 × ULN with development of new pathological Q waves according to the SCAI definition [16]. The secondary outcomes were other complications, such as slow flow just after RA, final TIMI flow grade ≤ 2, vessel perforation (type III) due to burr, and in-hospital death (irrespective of procedural complications).

The study was approved by the institutional review board of the Saitama Medical Center, Jichi Medical University (S22-125), and written informed consent was waved by the institutional review board of the Saitama Medical Center, Jichi Medical University, because of the retrospective study design. All procedures were in accordance with the Declaration of Helsinki. All methods were performed in accordance with relevant guidelines and regulations.

In most cases, RA was selected as the primary RA strategy, defined as RA before any attempt of balloon dilatation, whereas in some cases, RA was selected as the secondary RA strategy, defined as RA after unsuccessful balloon dilatation or unsuccessful balloon delivery. In the primary RA strategy, RA was performed using standard techniques. The lesion was crossed with a 0.014-inch conventional guidewire. Of 177 lesions, intravascular imaging including intravascular ultrasound (IVUS) or optical coherent tomography (OCT) before RA was tried in 129 lesions (72.9%). However, intravascular imaging devices could not cross the lesion in 65 lesions (36.7% of all lesions, 50.4% of tried lesions). Thus, intravascular imaging before RA was not available in 113 lesions (63.8% of all lesions). After intravascular imaging, a 0.014-inch conventional guidewire was exchanged for a 0.009-inch RotaWire floppy or RotaWire extra-support guidewire using a microcatheter. The RA burr was subsequently advanced over the wire to a position proximal to the lesion. Blood pressure (BP) and heart rate were recorded immediately before RA. The initial rotational speed was set within the conventional range (140,000–190,000 rpm) with the burr proximal to the lesion, and several lesions were randomly allocated to 140,000 rpm or 190,000 rpm [15]. The burr was activated and moved forward with a slow pecking motion. Each run time was < 30 s, and care was taken to avoid a decrease in rotational speed > 5000 rpm. Total run time was defined as the sum of each run time (high-speed mode). Total run time does not include either the interval time between the sessions or halftime. Furthermore, total run time does not include the time when the dynaglide mode is activated. The initial burr-size was either 1.25 mm, 1.5 mm, or 1.75 mm. After the burr passed the lesion, the burr was removed using the dynaglide mode or trapping balloon technique [17]. The presence of coronary flow was confirmed by injecting sufficient contrast medium immediately after the burr had been removed. An intra-aortic balloon pump (IABP) was inserted via a femoral artery before RA in high-risk cases such as those with severe left-ventricular dysfunction, unprotected left main stenosis, or severe 3-vessel disease.

Halftime was defined as at least one long break during RA, in which an operator pulled out the Rotablator system from the guide catheter before crossing the lesion. An operator needs to pull out the Rotablator system from the guide catheter when the burr-size down or the exchange of RotaWire is performed. Therefore, the lesions which required the burr-size down or the exchange of RotaWire before crossing the lesion were included in the halftime group. Furthermore, the lesions which required a long break (pull out the Rotablator system from the guide catheters) without burr-size down or exchange of RotaWire were also included in the halftime group. The definitions of hypertension, diabetes mellitus, and dyslipidemia are described elsewhere [7]. Estimate glomerular filtration rate (eGFR) was calculated using the MDRD formula (18). ACS was defined as ST-segment elevation MI, non-ST-segment elevation MI, or unstable angina [15]. The reference diameter and lesion length were calculated by quantitative coronary angiography. Offline, computer-based software QAngio XA 7.3 (MEDIS Imaging Systems, Leiden, The Netherlands) was used for quantitative coronary angiography. Calcification was identified as readily apparent radiopacities within the vascular wall at the site of the stenosis, and was classified as severe (radiopacities noted without cardiac motion before contrast injection generally compromising both sides of the arterial lumen) [19]. The burr-to-artery ratio was defined as the burr size divided by the reference diameter.

## Statistical analysis

Data are presented as a percentage for categorical variables, a mean ± standard deviation (SD) for normally distributed continuous variables, or a median (Q2) and inter-quartile range (Q1 -Q3) for non-normally distributed continuous variables. The Wilk–Shapiro test was performed to determine if the continuous variables were normally distributed. Normally distributed continuous variables were compared between the 2 groups using Student’s t test. Otherwise, continuous variables were compared using a Mann–Whitney U test. Categorical data were compared using a Fisher’s exact test. One-to-one propensity-score matching was performed as supplemental analysis. Halftime RA was set as a dependent variable, whereas parameters that were clinically relevant for complications in RA, such as age, male sex, estimated GFR, culprit lesion in acute coronary syndrome, reference diameter, and lesion length, were set as independent variables [7, 20, 21]. Because the number of the halftime RA group was 29, we limited independent variables as above 6 variables. For matching, the match tolerance was set as a width of 0.25 multiplied by the SD of the propensity score distribution [10, 22]. Case–control matching found 27 matches with maximizing matching performance. All reported P values were determined by two-sided analysis, and *P*-values < 0.05 were considered significant. All analyses were performed with IBM SPSS statistics version 25 (Chicago, IL, USA).

## Results

**Fig. 1:**
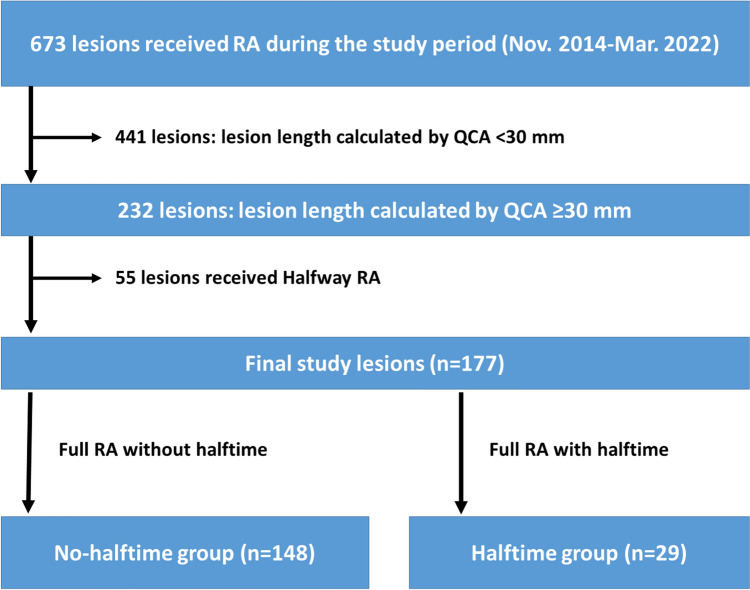
Study flowchart, *RA* = rotational atherectomy, *QCA* = quantitative coronary angiography

**Fig. 2:**
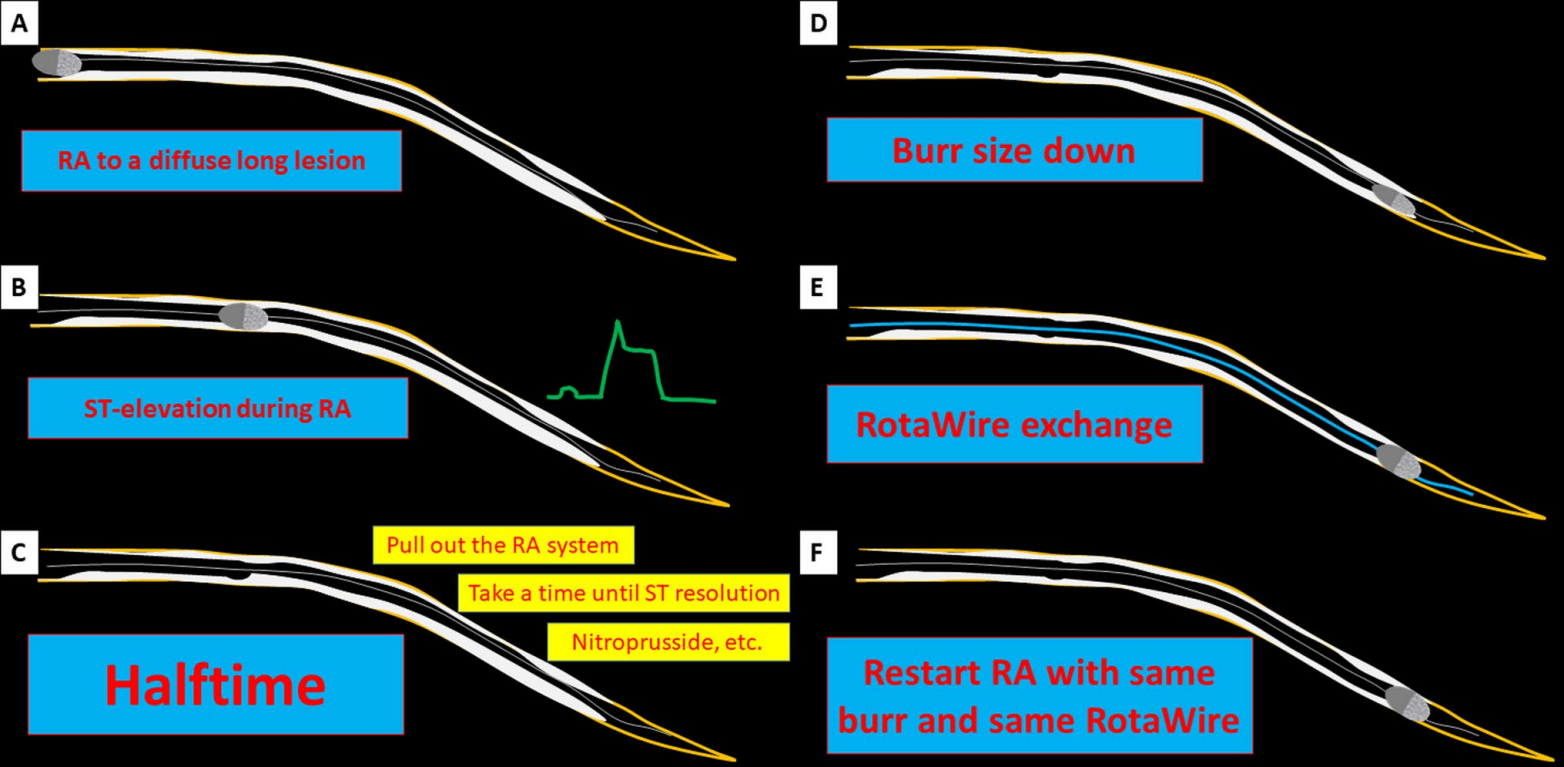
Representative illustration of halftime rotational atherectomy, Panel A. Rotational atherectomy (RA) to a diffuse long lesion is performed. Panel B. A marked ST-segment elevation is observed during RA. The burr is in the middle of the lesion. Panel C. The RA system including the burr is pulled out from the guide catheter. An operator takes a halftime until ST resolution. Intracoronary vasodilators such as nitroprusside can be injected. If coronary flow is fully recovered, an operator can select different approaches (Panels D, E, F). Panel D. The burr-size down is a standard approach for diffuse long calcified lesion. Panel E. The exchange of RotaWire is also a standard approach for diffuse long calcified lesion. Panel F. The restarting RA with same burr and same RotaWire is a unique approach for diffuse long calcified lesion. This approach does not require additional cost

Of 673 lesions, we excluded 441 lesions, in which the lesion length was less than 30 mm. Then, we excluded 55 lesions, in which halfway RA was performed. The final study consisted of 177 lesions. The lesions were classified into a halftime group (*n* = 29) and a no-halftime group (*n* = 148) according to the presence or absence of halftime. The study flowchart is shown in Fig. 1, and the concept of halftime RA is illustrated in Fig. 2.

Four different approaches were performed in the halftime group. The breakdown of the halftime group was as follows: [1] the burr-size down (*n* = 10), [2] the exchange of RotaWire (*n* = 6), [3] the combination of burr-size down and exchange of RotaWire (*n* = 2), and [4] the restarting RA with same burr-size and same RotaWire (*n* = 11). The comparison of patient and lesion characteristics between the no-halftime group and the halftime group is shown in Table 1. The patient characteristics except angiotensin-converting enzyme inhibitors/angiotensin receptor blockers treatment were similar between the 2 groups. The reference diameter was significantly smaller in the halftime group than in the no-halftime group, whereas the lesion length was similar between the 2 groups.

**Table 1:** Comparison of patients and lesions characteristics between the no-halftime group and the halftime group

	All (*n* = 177)	No-halftime group (*n* = 148)	Halftime group (*n* = 29)	*p* value
Patient characteristics				
Age (years)	75.0 (68.0–79.5)	75.0 (68.3–79.0)	74 (67.5–80.5)	0.596
Men—*n*, (%)	128 (72.3)	105 (70.9)	23 (79.3)	0.496
Overweight (BMI ≥ 25 kg/m^2^)—*n*, (%)	56 (31.6)	44 (29.7)	12 (41.4)	0.275
Hypertension—*n*, (%)	172 (97.2)	143 (96.6)	29 (100)	0.593
Diabetes mellitus—*n*, (%)	99 (55.9)	80 (54.1)	19 (65.5)	0.309
Hyperlipidemia—*n*, (%)	161 (91.0)	135 (91.2)	26 (89.7)	0.729
Current smoker—*n*, (%)	28 (15.9) (n = 176)	23 (15.5)	5 (17.9) (n = 28)	0.779
Chronic renal failure (creatinine > 2 mg/dl)—*n*, (%)	52 (29.4)	46 (31.1)	6 (20.7)	0.373
Estimated GFR (mL/min/1.73m^2^)	66.5 (16.5–94.0)	65.2 (13.1–92.8)	76.6 (41.8–102.3)	0.091
Left-ventricular ejection fraction (%)	58.0 (44.2–66.0) (n = 138)	58.5 (47.1–66.9) (n = 112)	57.9 (37.6–63.8) (n = 26)	0.238
Chronic renal failure on hemodialysis—*n*, (%)	45 (25.4)	40 (27.0)	5 (17.2)	0.353
History of hospitalization caused by heart failure – n, (%)	39 (22.0)	30 (20.3)	9 (31.0)	0.223
Statin treatment—*n*, (%)	158 (89.3)	132 (89.2)	26 (89.7)	1.000
ACE inhibitors/ARBs treatment—*n*, (%)*	125 (70.6)	100 (67.6)	25 (86.2)	0.047
Beta blockers treatment—*n*, (%)	139 (78.5)	115 (77.7)	24 (82.8)	0.629
Lesion characteristics				
Culprit lesion in acute coronary syndrome—*n*, (%)	34 (19.2)	27 (18.2)	7 (24.1)	0.448
Culprit lesion in acute coronary syndrome with visible thrombus- *n*, (%)	0	0	0	-
Chronic total occlusion – *n*, (%)	10 (5.6)	9 (6.1)	1 (3.4)	1.000
In-stent lesion – *n*, (%)	8 (4.5)	8 (5.4)	0	0.356
Target coronary artery				0.240
Left main- left anterior descending artery—*n*, (%)	135 (76.3)	116 (78.4)	19 (65.5)	
Left circumflex artery—*n*, (%)	6 (3.4)	5 (3.4)	1 (3.4)	
Right coronary artery—*n*, (%)	36 (20.3)	27 (18.2)	9 (31.0)	
Specific target coronary artery				
Ostial left main – *n*, (%)	0	0	0	^
Ostial left anterior descending artery – *n*, (%)	27 (15.3)	22 (14.9)	5 (17.2)	0.778
Ostial left circumflex artery – *n*, (%)	1 (0.6)	1 (0.7)	0	1.000
Ostial right coronary artery—n, (%)	8 (4.5)	5 (3.4)	3 (10.3)	0.125
Any ostial lesion – *n*, (%)	36 (20.3)	28 (18.9)	8 (27.6)	0.315
Reference diameter (mm)	2.09 (1.83–2.51)	2.17 (1.89–2.59)	1.82 (1.70–2.06)	0.002
Lesion length (mm)	40.67 (34.72–50.04)	40.96 (34.99–49.76)	40.22 (34.12–52.71)	1.000
Lesion angle				0.903
Mild angulation (< 30º)	93 (52.5)	79 (53.4)	14 (48.3)	
Moderate angulation (30-60º)	70 (39.5)	57 (38.5)	13 (44.8)	
Severe angulation (≥ 60º)	14 (7.9)	12 (8.1)	2 (6.9)	
Angiographically severe calcification	177 (100)	148 (100)	29 (100)	-
Pre-procedural TIMI-flow grade ≤ 2	30 (16.9)	23 (15.5)	7 (24.1)	0.281

Data are expressed as median and inter-quartile range or number (percentage). A Mann–Whitney U test was used for continuous variables, and a Fisher exact test was used for categorical variables, *GFR* glomerular filtration rate, *TIMI* Thrombolysis in myocardial infarction, *ACE* *inhibitors*  angiotensin-converting enzyme inhibitors, *ARBs*  angiotensin II receptor blockers, *One patient received angiotensin receptor neprilysin inhibito,

The comparison of procedural characteristics between the 2 groups is shown in Table 2. The number of used burrs was significantly higher in the halftime group than in the no-halftime group. The initial and final burr-to-artery ratio was significantly higher in the halftime group than in the no-halftime group. The total run time was significantly longer in the halftime group than in the non-halftime group. Final procedures were similar between the 2 groups.

**Table 2:** Comparison of procedural characteristics between the no-halftime group and the halftime group

	All (n = 177)	No-halftime group (n = 148)	Halftime group (n = 29)	*p* value
Primary RA strategy – n, (%)	149 (84.2)	124 (83.8)	25 (86.2)	1.000
Guiding catheter size and system				0.576
6Fr—n, (%)	7 (4.0)	5 (3.4)	2 (6.9)	
7Fr—n, (%)	160 (90.4)	134 (90.5)	26 (89.7)	
8Fr—n, (%)	10 (5.6)	9 (6.1)	1 (3.4)	
Intra-aortic balloon pump support—n, (%)	17 (9.6)	16 (10.8)	1 (3.4)	
RotaWire floppy as initial RA guidewire	139 (78.5)	120 (81.1)	19 (65.5)	0.082
Number of used burrs				0.001
1	136 (76.8)	122 (82.4)	14 (48.3)	
2	40 (22.6)	26 (17.6)	14 (48.3)	
3	1 (0.6)	0	1 (3.4)	
Initial burr size				< 0.001
1.25-mm	70 (39.5)	67 (45.3)	3 (10.3)	
1.5-mm	104 (58.8)	78 (52.7)	26 (89.7)	
1.75-mm	3 (1.7)	3 (2.0)	0	
Final burr size				0.721
1.25-mm	69 (39.0)	59 (39.9)	10 (34.5)	
1.5-mm	78 (44.1)	63 (42.6)	15 (51.7)	
1.75-mm	14 (7.9)	13 (8.8)	1 (3.4)	
2.0-mm	16 (9.0)	13 (8.8)	3 (10.3)	
Initial burr-to-artery ratio	0.66 ± 0.16	0.64 ± 0.15	0.78 ± 0.17	< 0.001
Final burr-to-artery ratio	0.67 (0.57–0.82)	0.66 (0.56–0.78)	0.74 (0.64–0.89)	0.011
Total run time (seconds)	78.0 (45.0–126.5)	71.5 (42.0–108.0)	133.0 (102.0–223.0)	< 0.001
Mean single run time (seconds)	12.0 (9.7–16.0)	12.0 (9.7–15.9)	11.6 (9.5–16.0)	0.784
Mean rotational speed (× 1000 rpm)	176.8 (171.4–180.4)	176.8 (168.5–180.3)	179.1 (174.5–182.9)	0.068
Maximum speed reduction during RA (rpm)	6000 (5000–10000) (n = 172)	6000 (4000–8750) (n = 144)	8000 (6000–11000) (n = 28)	0.003
Systolic blood pressure just before RA (mm Hg)	147 (134–168)	147 (133–166)	153 (132–173)	0.623
Diastolic blood pressure just before RA (mm Hg)	75 (68–84)	75 (69–84)	74 (62–85)	0.382
Heart rate just before RA (per minute)	69 (62–78)	70 (61–79)	67 (62–73)	0.286
Final procedure				1.000
RA + balloon including drug-coating balloon—n, (%)	9 (5.1)	8 (5.4)	1 (3.4)	
RA + drug-eluting stent—n, (%)	162 (91.5)	134 (90.5)	28 (96.6)	
RA + drug-eluting stent + drug-coating balloon – n, (%)	3 (1.7)	3 (2.0)	0	
RA + covered stent for perforation—n, (%)¶	3 (1.7)	3 (2.0)	0	

Data are expressed as median and inter-quartile range or number (percentage). A Mann–Whitney U test was used for continuous variables, and a Fisher exact test was used for categorical variables. A case did not have perforation after RA, but required covered stent after stent implantation

The comparison of complications and outcomes between the 2 groups is shown in Table 3. The incidence of slow flow just after RA was higher in the halftime group than in the no-halftime group without reaching statistical significance. Although CK and CK–MB at the next day of RA was significantly higher in the halftime group than in the no-halftime group, neither periprocedural MI nor in-hospital death was observed in the halftime group.

**Table 3:** Comparison of complications and outcomes between the no-halftime group and the halftime group

	All (n = 177)	No-halftime group (n = 148)	Halftime group (n = 29)	*p* value
Complications and outcomes				
Slow flow just after RA – *n*, (%)	42 (23.7)	31 (20.9)	11 (37.9)	0.058
Final TIMI flow grade ≤ 2 – *n*, (%)	1 (0.6)	1 (0.7)	0	1.000
Vessel perforation (Type III) due to burr – *n*, (%)	2 (1.1)	2 (1.4)	0	1.000
Periprocedural myocardial infarction – *n*, (%)	1 (0.6)	1 (0.7)	0	1.000
Creatinine kinase at the next day of RA (U/L)	106 (66–229)	99 (59 – 216)	156 (97 – 308)	0.021
Creatinine kinase-myocardial band at the next day of RA (U/L)	6 (3 – 18)	5 (3 – 15)	15 (8 – 24)	< 0.001
In-hospital death (irrespective of procedural complications)	1 (0.6)	1 (0.7)	0	1.000

Data are expressed as median and inter-quartile range or number (percentage). A Mann–Whitney U test was used for continuous variables, and a Fisher exact test was used for categorical variables

The comparison of patient and lesion characteristics between the matched no-halftime group and the matched halftime group is shown in Supplemental Table 1. The reference diameter as well as the lesion length were similar between the matched 2 groups. The comparison of procedural characteristics between the matched 2 groups is shown in Supplemental Table 2. The number of used burrs was significantly higher in the matched halftime group than in the matched no-halftime group. The comparison of complications and outcomes between the matched 2 groups is shown in Supplemental Table 3. The incidence of slow flow just after RA was similar between the matched 2 groups.

## Discussion

We included 177 diffuse severely calcified lesions that underwent RA, and divided them into the no-halftime group (*n* = 148) and the halftime group (*n* = 29). The breakdown of the halftime group was as follows: [1] the burr-size down (*n* = 10), [2] the exchange of RotaWire (*n* = 6), [3] the combination of burr-size down and exchange of RotaWire (*n* = 2), and [4] the restarting RA with same burr-size and same RotaWire (*n* = 11). The reference diameter was significantly smaller in the halftime group than in the no-halftime group, whereas the lesion length was similar between the 2 groups. Total run time was significantly longer in the halftime group than in the no-halftime group. Although CK and CK–MB at the next day of RA was significantly higher in the halftime group than in the no-halftime group, neither periprocedural MI nor in-hospital death was observed in the halftime group. Our results suggest the possibility of halftime RA as an alternative option for diffuse severely calcified lesions.

Among halftime RA approaches, the burr-size down is a standard approach when an operator feels difficulty to cross the diffuse long lesions [8, 9]. Although this approach is well established among RA experts, there are no clinical studies that investigate the safety and efficacy of the burr-size down approach. In our cohort, approximately one-third of the halftime group used this approach, which suggests that the burr-size down may be useful to ablate the whole lesion without increasing complications. The exchange of RotaWire is also a well-established approach when an operator feels difficulty to cross the diffuse long lesions. There are 2 ways in the exchange of RotaWire. One is from RotaWire floppy to RotaWire extra-support, and the other is from RotaWire extra-support to RotaWire floppy. The former way is more commonly used than the latter way among RA experts. In our results, all cases with exchange of RotaWire adopted the former way. The exchange of RotaWire would modify the guidewire bias, which may facilitate the advancement of the burr. The combination of the burr-size down and the exchange of RotaWire can be the last resort for an uncrossable diffuse long lesion. It is natural for RA experts to select this approach, if the second burr or the second RotaWire does not work. The fourth approach is to restart RA with same burr and same RotaWire. This approach has not been recognized as an option for the diffuse long lesions. The concept of this approach is to take a halftime to stabilize the situation, and then restart RA with the same system if coronary flow is fully recovered. In our cohort, more than one-third of the halftime group used this approach. This approach does not require additional cost. If this approach does not work, operators can consider other approaches such as burr-size down. As compared to the former 3 halftime approaches, this approach (restart RA with the same burr and same RotaWire) is a novel halftime RA. The details of each halftime approach are summarized in Table 4.

**Table 4:** The details of each halftime approach

	Burr-size down (*n* = 10)	Exchange of RotaWire (*n* = 6)	Combination of the burr-size down and the exchange of RotaWire (*n* = 2)	Restarting RA with same burr size and same RotaWire (*n* = 11)
Notes	Standard approach when an operator feels difficulty to cross the diffuse long lesions	All 6 cases used a RotaWire floppy as initial RotaWire. Then, exchanged to Extra-Support Wire	The combination of the burr-size down and the exchange of RotaWire would be the last resort for an uncrossable diffuse long lesion	This approach has not been recognized for the diffuse long lesions. Just take a halftime to stabilize the situation, and then restart RA with the same system
Strength	This approach is well-established among RA experts	This approach is also established among RA experts	It is natural to select this approach, if the second burr or the second RotaWire does not work	This approach does not require additional cost. If this approach does not work, an operator can consider other approaches such as burr-size down
Weakness	The second burr requires additional cost	The second RotaWire requires additional cost	The second burr and RotaWire require additional cost	The same system (burr and RotaWire) may not work after halftime
Rationale	The second (small) burr would require less energy than the first burr to cross the lesion	Different guidewire bias by the second RotaWire would work for crossing	Combination of burr-size down and exchange of RotaWire	If taking halftime was decided by ST-segment elevation or transient slow flow rather than the lesion’s hardness, we could restart RA without changing the burr size or RotaWire after ST-segment resolution or recovery of slow flow

The most important step in halftime RA is to stop RA when a marked ST-segment elevation is observed during RA. Unlike slow flow in the treatment of acute coronary syndrome, slow flow during RA progress gradually. If mild slow flow (TIMI flow grade 2) is properly addressed, the progression to severe slow flow (TIMI flow grade ≤ 1) is uncommon. However, if an operator ignores the signs of mild slow flow such as chest pain or ST-segment elevation, the progression to severe slow flow can occur, which results in periprocedural MI or subsequent procedure-related death. Since ST-segment elevation precedes slow flow in most cases, the decision-making to take a halftime highly depends on the appearance of marked or sustained ST-segment elevation during RA. The second important step in halftime RA is to pull out the RA system from the guide catheter. Because the diameter (1.43 mm) of the drive shaft sheath of Rotablator is larger than that of the small burr, the presence of the RA system within coronary artery would impede the recovery from slow flow. Since the drive shaft sheath of Rotablator is radiolucent, it is difficult for operators to recognize the position of the derive shaft sheath during RA. Furthermore, when intracoronary vasodilators such as nitroprusside are injected, the presence of the RA system would prevent those vasodilators from reaching to the distal coronary vascular bed. The third step in halftime RA is to wait for the recovery of coronary flow. Although the complete ST resolution may be difficult to achieve, a TIMI-grade 3 coronary flow is necessary to restart RA. Operators can spend the waiting time by performing intravascular imaging such as intravascular ultrasound. Finally, operators can select an appropriate approach among 4 different approaches (the burr-size down, the exchange of RotaWire, the combination of the burr-size down and the exchange of RotaWire, and restarting RA with same burr and same RotaWire).

Clinical implications of the present study should be noted. Because the coronary calcification represents the advancement of atherosclerosis, patients who undergo RA often have other comorbidities. RA to the diffuse severely calcified lesion may be performed in the context of complex and high-risk intervention in indicated patients (CHIP) [23]. Since severe slow flow can be fatal in CHIP, the option to prevent severe slow flow is of utmost importance. Our group previously proposed halfway RA especially for severely angulated lesions [10, 11]. Halfway RA, which is a combination of RA and balloon dilatation, is an option to prevent severe complications, such as vessel perforation or burr entrapment [10, 11]. However, since halfway RA mostly ablates the proximal half of the lesion, halfway RA may not work for some diffuse long lesions, especially severe calcification at the distal half of the lesions. Halftime RA, which is intended for ablating the whole lesion, may be an alternative option to prevent severe slow flow for any types of severely calcified lesions.

Since this study is a single-center, retrospective, observational study, there is a risk of patient selection bias and group selection bias. Because we experienced typical cases of halftime RA (restarting RA with same burr and same RotaWire) in the middle of the study period, we selected halftime RA more frequently after those cases, which can be a selection bias. Although care was taken to avoid a decrease in rotational speed > 5000 rpm, a decrease of > 5000 rpm was frequently observed in this study lesions. This means that our care might not be enough to prevent a decrease of > 5000 rpm or a decrease of > 5000 rpm might be unavoidable for diffuse long severely calcified lesions. Furthermore, although total run time did not include either the interval time between the sessions or halftime, total run time was significantly longer in the halftime than in the no-halftime group. It is unclear whether halftime RA strategy increases the ablation time itself or lesions in the halftime group were more severely calcified lesions. We did not define long break or halftime by the actual length of a break. However, it takes at least several minutes to pull out the Rotablator system from the guide catheter and reinsert the Rotablator system into the guide catheter. Although we conducted a propensity-score matching to adjust clinical background between the no-halftime group and the halftime group, the number of generated pairs was small. This preliminary study was conducted to generate a hypothesis that halftime RA can be a safe option for diffuse severely calcified lesions. Future studies are warranted to assess the safety and efficacy of halftime RA using a large sample size.

## Conclusions

Although CK and CK–MB at the next day of RA was significantly higher in RA with halftime than in RA without halftime, neither periprocedural MI nor in-hospital death was observed in RA with halftime. Halftime RA may be an alternative option for diffuse severely calcified lesions.

### Supplementary Information

Below is the link to the electronic supplementary material.

Supplementary file1 (DOCX 41 KB)

## Data Availability

All data are available from the corresponding author on reasonable request.
