# Supplementary material for: Halftime rotational atherectomy: a unique concept for diffuse long severely calcified lesions
Source: Cardiovasc Interv Ther. 2023 Nov 10;39(1):18–27. doi: 10.1007/s12928-023-00968-1 (PMC10764388; doi:10.1007/s12928-023-00968-1)
Supplement: Supplementary file 1 — Supplementary file1 (DOCX 41 KB) [file 12928_2023_968_MOESM1_ESM.docx]

**Supplemental Table 1. Comparison of patients and lesions characteristics between the matched no-halftime group and the matched halftime group**

|  | All  (n = 54) | Matched no-halftime group  (n = 27) | Matched halftime group  (n = 27) | *p* value |
| --- | --- | --- | --- | --- |
| Patient characteristics |  |  |  |  |
| Age (years) | 72.5 (68.0 – 79.0) | 72.0 (68.0 – 78.0) | 74.0 (68.0 -81.0) | 0.782 |
| Men - n, (%) | 42 (77.8) | 21 (77.8) | 21 (77.8) | 1.000 |
| Overweight (BMI ≥25 kg/m^2^) - n, (%) | 19 (35.2) | 7 (25.9) | 12 (44.4) | 0.254 |
| Hypertension - n, (%) | 51 (94.4) | 24 (88.9) | 27 (100) | 0.236 |
| Diabetes mellitus - n, (%) | 34 (63.0) | 16 (59.3) | 18 (66.7) | 0.779 |
| Hyperlipidemia - n, (%) | 51 (94.4) | 27 (100) | 24 (88.9) | 0.236 |
| Current smoker - n, (%) | 9 (17.0) (n=53) | 4 (14.8) | 5 (19.2) (n=26) | 0.728 |
| Chronic renal failure (creatinine >2mg/dl) - n, (%) | 9 (16.7) | 3 (11.1) | 6 (22.2) | 0.467 |
| Estimated GFR (mL/min/1.73m^2^) | 71.8±33.8 | 74.8±28.9 | 68.8±38.4 | 0.515 |
| Left ventricular ejection fraction (%) | 61.0 (52.8 – 67.0) (n=45) | 65.0 (56.0 – 70.5) (n=21) | 58.5 (39.9 – 63.9) (n=24) | 0.031 |
| Chronic renal failure on hemodialysis - n, (%) | 6 (11.1) | 1 (3.7) | 5 (18.5) | 0.192 |
| History of hospitalization caused by heart failure – n, (%) | 14 (25.9) | 6 (22.2) | 8 (29.6) | 0.757 |
| Statin treatment - n, (%) | 51 (94.4) | 27 (100) | 24 (88.9) | 0.236 |
| ACE inhibitors/ARBs treatment - n, (%) | 40 (74.1) | 17 (63.0) | 23 (85.2) | 0.119 |
| Beta blockers treatment - n, (%) | 44 (81.5) | 22 (81.5) | 22 (81.5) | 1.000 |
|  |  |  |  |  |
| Lesion characteristics |  |  |  |  |
| Culprit lesion in acute coronary syndrome - n, (%) | 11 (20.4) | 5 (18.5) | 6 (22.2) | 1.000 |
| Culprit lesion in acute coronary syndrome with visible thrombus- n, (%) | 0 | 0 | 0 | - |
| Chronic total occlusion – n, (%) | 1 (1.9) | 0 (0) | 1 (3.7) | 1.000 |
| In-stent lesion – n, (%) | 0 | 0 | 0 | - |
| Target coronary artery |  |  |  | 0.142 |
| Left main- left anterior descending artery - n, (%) | 43 (79.6) | 24 (88.9) | 19 (70.4) |  |
| Left circumflex artery - n, (%) | 2 (3.7) | 1 (3.7) | 1 (3.7) |  |
| Right coronary artery - n, (%) | 9 (16.7) | 2 (7.4) | 7 (25.9) |  |
| Specific target coronary artery |  |  |  |  |
| Ostial left main – n, (%) | 0 | 0 | 0 | - |
| Ostial left anterior descending artery – n, (%) | 8 (14.8) | 3 (11.1) | 5 (18.5) |  |
| Ostial left circumflex artery – n, (%) | 0 | 0 | 0 | - |
| Ostial right coronary artery - n, (%) | 3 (5.6) | 1 (3.7) | 2 (7.4) | 1.000 |
| Any ostial lesion – n, (%) | 11 (20.4) | 4 (14.8) | 7 (25.9) | 0.501 |
| Reference diameter (mm) | 1.89 (1.70 – 2.32) | 1.97 (1.64 – 2.35) | 1.84 (1.70 – 2.08) | 0.622 |
| Lesion length (mm) | 39.59 (34.56 – 50.68) | 39.62 (34.64 – 48.89) | 38.81 (33.64 – 52.62) | 0.924 |
| Lesion angle |  |  |  | 0.721 |
| Mild angulation (<30º) | 26 (48.1) | 14 (51.9) | 12 (44.4) |  |
| Moderate angulation (30-60º) | 23 (42.6) | 10 (37.0) | 13 (48.1) |  |
| Severe angulation (≥60º) | 5 (9.3) | 3 (11.1) | 2 (7.4) |  |
| Angiographically severe calcification | 54 (100) | 27 (100) | 27 (100) | - |
| Pre-procedural TIMI-flow grade ≤2 | 11 (20.4) | 4 (14.8) | 7 (25.9) | 0.501 |

Data are expressed as median and inter-quartile range or number (percentage). A Mann-Whitney U test was used for continuous variables, and a Fischer exact test was used for categorical variables. Abbreviations: GFR = glomerular filtration rate, TIMI = Thrombolysis in myocardial infarction, ACE inhibitors = angiotensin converting enzyme inhibitors, ARBs = angiotensin II receptor blockers.

**Supplemental Table 2. Comparison of procedural characteristics between the matched no-halftime group and the matched halftime group**

|  | All  (n = 54) | Matched no-halftime group  (n = 27) | Matched halftime group  (n = 27) | *p* value |
| --- | --- | --- | --- | --- |
| Primary RA strategy – n, (%) | 47 (87.0) | 24 (88.9) | 23 (85.2) | 1.000 |
| Guiding catheter size and system |  |  |  | 1.000 |
| 6Fr - n, (%) | 1 (1.9) | 0 (0) | 1 (3.7) |  |
| 7Fr - n, (%) | 50 (92.6) | 25 (92.6) | 25 (92.6) |  |
| 8Fr - n, (%) | 3 (5.6) | 2 (7.4) | 1 (3.7) |  |
| Intra-aortic balloon pump support - n, (%) | 5 (9.3) | 4 (14.8) | 1 (3.7) | 0.351 |
| RotaWire floppy as initial RA guidewire | 43 (79.6) | 24 (88.9) | 19 (70.4) | 0.175 |
| Number of used burrs |  |  |  | <0.001 |
| 1 | 38 (70.4) | 26 (96.3) | 12 (44.4) |  |
| 2 | 15 (27.8) | 1 (3.7) | 14 (51.9) |  |
| 3 | 1 (1.9) | 0 (0) | 1 (3.7) |  |
| Initial burr size |  |  |  | <0.001 |
| 1.25-mm | 28 (51.9) | 25 (92.6) | 3 (11.1) |  |
| 1.5-mm | 26 (48.1) | 2 (7.4) | 24 (88.9) |  |
| 1.75-mm | 0 (0) | 0 (0) | 0 (0) |  |
| Final burr size |  |  |  | <0.001 |
| 1.25-mm | 34 (63.0) | 24 (88.9) | 10 (37.0) |  |
| 1.5-mm | 15 (27.8) | 2 (7.4) | 13 (48.1) |  |
| 1.75-mm | 2 (3.7) | 1 (3.7) | 1 (3.7) |  |
| 2.0-mm | 3 (5.6) | 0 (0) | 3 (11.1) |  |
| Initial burr-to-artery ratio | 0.71±0.18 | 0.65±0.17 | 0.77±0.17 | 0.012 |
| Final burr-to-artery ratio | 0.71±0.20 | 0.66±0.19 | 0.76±0.20 | 0.050 |
| Total run time (seconds) | 126.5 (98.5 – 182.0) | 119.0 (97.0 – 138.0) | 133.0 (110.0 – 223.0) | 0.062 |
| Mean single run time (seconds) | 15.6±4.8 | 18.5±3.1 | 12.7±4.5 | <0.001 |
| Mean rotational speed (x 1000 rpm) | 176.1 (157.0 – 182.7) | 158.6 (138.2 – 185.3) | 176.5 (174.1 – 180.3) | 0.068 |
| Maximum speed reduction during RA (rpm) | 6000 (5000 – 10000) (n=51) | 6000 (4500 – 7000) (n=25) | 7500 (6000 – 10500) (n=26) | 0.003 |
| Systolic blood pressure just before RA (mm Hg) | 147±26 | 141±22 | 154±28 | 0.072 |
| Diastolic blood pressure just before RA (mm Hg) | 74±11 | 75±9 | 74±13 | 0.750 |
| Heart rate just before RA (per minute) | 67 (61 – 74) | 69 (60 – 77) | 66 (61 – 73) |  |
| Final procedure |  |  |  | 0.491 |
| RA + drug-eluting stent - n, (%) | 52 (96.3) | 25 (92.6) | 27 (100) |  |
| RA + covered stent for perforation - n, (%) | 2 (3.7) | 2 (7.4) | 0 (0) |  |

Data are expressed as median and inter-quartile range or number (percentage). A Mann-Whitney U test was used for continuous variables, and a Fischer exact test was used for categorical variables.

**Supplemental Table 3. Comparison of complications and outcomes between the matched no-halftime group and the matched halftime group**

|  | All  (n = 54) | Matched no-halftime group  (n = 27) | Matched halftime group  (n = 27) | *p* value |
| --- | --- | --- | --- | --- |
| Complications and outcomes |  |  |  |  |
| Slow flow just after RA – n, (%) | 23 (42.6) | 13 (48.1) | 10 (37.0) | 0.583 |
| Final TIMI flow grade ≤2 – n, (%) | 0 (0) | 0 (0) | 0 (0) | - |
| Vessel perforation (Type III) due to burr – n, (%) | 2 (3.7) | 2 (7.4) | 0 (0) | 0.491 |
| Periprocedural myocardial infarction – n, (%) | 1 (1.9) | 1 (3.7) | 0 (0) | 1.000 |
| Creatinine kinase at the next day of RA (U/L) | 136 (71 – 298) | 105 (59 – 351) | 136 (96 – 243) | 0.483 |
| Creatinine kinase-myocardial band at the next day of RA (U/L) | 11 (3 – 21) | 5 (1 – 16) | 15 (8 – 23) | 0.021 |
| In-hospital death (irrespective of procedural complications) | 1 (1.9) | 1 (3.7) | 0 (0) | 1.000 |

Data are expressed as median and inter-quartile range or number (percentage). A Mann-Whitney U test was used for continuous variables, and a Fischer exact test was used for categorical variables.
